# RNA sequencing reveals candidate genes and polymorphisms related to sperm DNA integrity in testis tissue from boars

**DOI:** 10.1186/s12917-017-1279-x

**Published:** 2017-11-28

**Authors:** Maren van Son, Nina Hårdnes Tremoen, Ann Helen Gaustad, Frøydis Deinboll Myromslien, Dag Inge Våge, Else-Berit Stenseth, Teklu Tewoldebrhan Zeremichael, Eli Grindflek

**Affiliations:** 1 Topigs Norsvin, 2317 Hamar, Norway; 2 Department of Natural Sciences and Technology, Inland Norway University of Applied Sciences, 2318 Hamar, Norway; 3 Centre for Integrative Genetics (CIGENE), Department of Animal and Aquacultural Sciences, Faculty of Biosciences, Norwegian University of Life Sciences, 1432 Ås, Norway

**Keywords:** Transcriptome profiling, Sperm DNA integrity, Differential expression

## Abstract

**Background:**

Sperm DNA is protected against fragmentation by a high degree of chromatin packaging. It has been demonstrated that proper chromatin packaging is important for boar fertility outcome. However, little is known about the molecular mechanisms underlying differences in sperm DNA fragmentation. Knowledge of sequence variation influencing this sperm parameter could be beneficial in selecting the best artificial insemination (AI) boars for commercial production. The aim of this study was to identify genes differentially expressed in testis tissue of Norwegian Landrace and Duroc boars, with high and low sperm DNA fragmentation index (DFI), using transcriptome sequencing.

**Results:**

Altogether, 308 and 374 genes were found to display significant differences in expression level between high and low DFI in Landrace and Duroc boars, respectively. Of these genes, 71 were differentially expressed in both breeds. Gene ontology analysis revealed that significant terms in common for the two breeds included extracellular matrix, extracellular region and calcium ion binding. Moreover, different metabolic processes were enriched in Landrace and Duroc, whereas immune response terms were common in Landrace only. Variant detection identified putative polymorphisms in some of the differentially expressed genes. Validation showed that predicted high impact variants in *RAMP2*, *GIMAP6* and three uncharacterized genes are particularly interesting for sperm DNA fragmentation in boars.

**Conclusions:**

We identified differentially expressed genes between groups of boars with high and low sperm DFI, and functional annotation of these genes point towards important biochemical pathways. Moreover, variant detection identified putative polymorphisms in the differentially expressed genes. Our results provide valuable insights into the molecular network underlying DFI in pigs.

**Electronic supplementary material:**

The online version of this article (10.1186/s12917-017-1279-x) contains supplementary material, which is available to authorized users.

## Background

Analysis of sperm parameters is important for predicting boar fertility and the outcome of artificial insemination (AI) in pig production. The classical way of evaluating sperm parameters is subjective scoring of viability, motility, concentration and morphology, to identify ejaculates with poor fertilization potential [1, 2]. However, this is insufficient for accurate prediction of the boar’s reproductive capacity, since the sperm cells must have additional qualities to fertilize the oocytes and since it is a subjective score. Combining several assays is suggested to better predict the fertility of an ejaculate [3]. For example, combining sperm morphology parameters and evaluation of DNA chromatin integrity has been found to be related to field fertility, as measured by farrowing rate in pigs [4].

During the last phase of spermatogenesis, spermiogenesis, the DNA of sperm cells is tightly packed by protamine and results in a condensed chromatin structure [5]. This leaves the DNA protected against degradation during transport through the male and female reproductive tracts until fertilization. Altered sperm chromatin structure is associated with DNA fragmentation and the degree of sperm DNA fragmentation is shown to be correlated to fertility in different species [4, 6–13]. This parameter is a much more objective marker of sperm quality and function than standard subjective microscopic evaluations [14, 15]. The sperm chromatin structure assay (SCSA) is a flow cytometry-based method that measures abnormal chromatin structure, as an increased acid-induced degradation of sperm DNA in situ [11]. More specifically, the acid denatures DNA at the sites of DNA breaks, which again reflects chromatin integrity status. The SCSA thereafter measures the relationship between double-stranded (i.e. condensed chromatin) and single-stranded (i.e. denatured) DNA for each sperm cell. This relationship is quantified by the DNA Fragmentation Index (DFI) [12]. Previous studies in pigs showed that DFI was significantly associated with litter size [8]. Moreover, DFI is found to be an important parameter for predicting normal development of the embryo [11, 16] and is also associated with abortion in humans [17].

Although the amount of sperm DFI is shown to influence fertility outcome, little is known about the underlying molecular mechanisms. Differentially expressed proteins have been identified in human seminal plasma and spermatozoa [18, 19]. Studies in humans have also showed that a truncated form of KIT tyrosine kinase, expressed in testis, causes higher amounts of DNA damage in sperm cells [20]. Moreover, depletion of excision repair cross-complementing gene 1 (*ERCC1*) and tumor suppressor gene *p53* in mouse testis resulted in increased DNA breaks in sperm cells [21]. Recent studies indicate that the main reason of DFI in sperm is apoptosis, likely triggered by an impairment of chromatin maturation in the testis and by oxidative stress during the transit in the male genital tract [22].

The goal of this study was to use transcriptome sequencing to examine differential gene expression in testis tissue of boars with high and low sperm DFI. Testis tissue was chosen because chromatin condensation and DNA packaging in sperm cells occurs during testicular spermatogenesis [5, 23]. The biological functions of the differentially expressed genes were also investigated and a search for putative polymorphisms in the differentially expressed genes was performed. The results obtained in this study highly contribute to the knowledge of the molecular mechanisms underlying DNA fragmentation.

## Methods

### Animals and phenotypes

The sperm DFI was determined in a total of 241 Landrace and 302 Duroc AI boars in this study. All the boars were housed individually in pens sized approximately two by three meters and fed the same commercial diet. Nine Landrace and eleven Duroc boars were selected for transcriptome profiling based on their extreme high/low DFI values (Table 1). The boars’ age at semen sample collection ranged from 221 to 1000 days (mean = 310 days, standard deviation (SD) = 84.5). The sperm-rich fraction of the ejaculates was collected with the “gloved hand technique” at the Norsvin AI center (Hamar, Norway), similar to other studies recently published [24, 25]. From each of the boars, samples from up to six different ejaculates were analyzed, and the mean of the measurements was used as the final score. The ejaculates were diluted to a concentration of 28 × 10^6^ spermatozoa per ml, according to the normal routines of the AI center at each date. The ejaculates were shipped as regular semen doses to commercial swine producers for the use within the next four days. From each individually diluted ejaculate, a sample of approximately 12 mL was transferred to a plastic tube. The samples were stored at 18 °C for 48 to 96 h depending on day of the week, before they were frozen in −80 °C until used for the DFI analysis. Boars were culled according to normal culling procedures at the AI station. From these boars, the testicle tissue samples were collected at the slaughter line. A piece of sample was collected from the middle part of one of the testicles, approximately 3 × 1.5 cm in size, immediately frozen in liquid N_2,_ and thereafter stored at −80 °C until used for RNA extraction.

**Table 1:** DFI measurements for the different boars used in this study

Group	Boar	n(ejaculates)	DFI mean	DFI SD
Landrace low	L1	2	0.62%	0.10%
	L2	1	0.73%	
	L3	1	0.82%	
	L4	3	1.50%	0.33%
	L5	4	1.52%	0.68%
Landrace high	L6	1	6.41%	
	L7	2	5.47%	1.69%
	L8	6	7.26%	5.11%
	L9	7	8.07%	3.85%
Duroc low	D1	1	1.05%	
	D2	2	1.07%	0.37%
	D3	2	1.08%	0.34%
	D4	1	1.11%	
	D5	3	1.13%	0.35%
Duroc high	D6	1	4.13%	
	D7	1	4.77%	
	D8	1	4.14%	
	D9	1	4.69%	
	D10	1	5.63%	
	D11	3	5.36%	3.64%

The number of ejaculates and mean DFI value with SD is presented for each boar

### DFI measurements

The SCSA protocol was performed using Cell Lab Quanta™ SC MPL (Beckman Coulter, Fullerton, CA, USA), equipped with a 22 mW argon laser with excitation at 488 nm, according to the procedure described by Evenson and Jost [13] with modifications [26]. The method is based on DNA staining properties of acridine orange (AO) which fluoresces green and red when binding to native dsDNA and denatured ssDNA, respectively. Frozen samples were thawed at 37 °C and diluted to a concentration of 2 × 10^6^ sperm cells/mL in TNE buffer (10 mM Tris-HCL, 0.15 M NaCl, 1 mM EDTA, pH 7.4) to a final volume of 200 μL. Immediately afterwards, 400 μL of acid detergent solution (0.38 M NaCl, 80 mM HCL, 0.1% (*w*/*v*) Triton X-100, pH 1.2) was added. After exactly 30 s, 1.2 mL of AO staining solution (0.6 μg/mL AO (A3568, Life Technologies, OR, USA) in a buffer containing 37 mM citric acid, 0.126 M Na_2_HPO_4_, 1.1 μM EDTA, 0.15 M NaCl, pH 6) was added, and the sample was further incubated at room temperature in the flow cytometer. The sample was run in setup mode until 3 min after addition of the acid detergent solution, and then the acquisition of data was started. For each sample, 5000 events were collected with a flow rate of ~200 events/s. Prior to the analysis, the flow cytometer was AO saturated by running an AO equilibration solution (1.2 mL AO staining solution and 400 μL acid detergent solution) through the system for 5 min. The green fluorescence was collected by a 525 nm band pass filter, while the red fluorescence was collected by a 670 nm long pass filter. Prior to analysis and after every 10th sample, a reference sample was thawed, prepared and analyzed in the same way as the experimental samples to ensure the stability of the instrument and the laser throughout the experiment. The X-mean channel value was set to 125 ± 5 and Y-mean channel value was set to 425 ± 5. To identify the spermatozoa, a combination of electronic volume (EV)- and side scatter (SS)- signals were used, as described by Standerholen et al. [27]. The percentage of red and green fluorescence was determined using the Cell Lab Quanta™ SC MPL Analysis software package (Beckman Coulter, Software Version 1.0 A). Based on the ratio of red/(red + green), the DFI-value was calculated.

### RNA extraction and sequencing

Total RNA for RNA sequencing was extracted from testicle tissue using the RNeasy Midi Kit from Qiagen according to the manufacturer’s instruction (Qiagen, CA, USA). Concentrations were measured using a NanoDrop ND-1000 Spectrophotometer (NanoDrop Technologies, DE, USA) and the RNA quality was examined by the 28S:18S rRNA ratio using the RNA 6000 Nano LabChip® Kit on 2100 Bioanalyzer (Agilent Technologies, CA, USA). All samples displayed a 260/280 ratio > 1.8 and RNA integrity numbers (RIN) >8.5. RNA sequencing was done using Illumina HiSeq 2000 by the Norwegian Sequencing Centre at Ullevål Hospital (http://www.sequencing.uio.no). and generated 50 basepair single end reads. TruSeq RNA v2 was used for non-stranded library preparation, V3 clustering and sequencing reagents were used according to manufacturer’s instructions. Sample amount of 2 μg RNA was used as input, and 4 min fragmentation at 94 °C was employed. Image analysis and base calling were performed using Illumina’s RTA software version 1.17.21.3. Reads were filtered to remove those with low base call quality using Illumina’s default chastity criteria. The FASTQC software was used for quality control of raw sequence data (http://www.bioinformatics.babraham.ac.uk/projects/fastqc). All reads had a per base sequence quality Phred score above 27 for all positions and were considered high quality. The data discussed in this publication have been deposited in NCBI’s Gene Expression Omnibus (GEO) [28] and are accessible through GEO Series accession number GSE74934.

### Differential expression

The high quality reads were mapped to the *Sus scrofa* genome build 10.2 using the software TopHat v.2.0.12 [29] and default parameters. The Picard AddOrReplaceReadGroups program (http://broadinstitute.github.io/picard/) was used to assign unique IDs to the files. Gene prediction coordinates (release 10.2.75) were obtained from the ENSEMBL web site (http://www.ensembl.org). Mapped reads were sorted and indexed using Samtools v.1.1 [30] and the software HTSeq [31] was used with the stranded = no option to calculate the number of reads mapped to each gene. The R software package “edgeR” v.3.2.4 from Bioconductor was used to analyze the data [32] [see Additional file 6 for code]. The breeds were analyzed separately and the boars were divided into “high” and “low” groups based on their DFI values. The package assumes that the data follow a negative binomial distribution and it uses raw counts without correcting for gene length as this bias is assumed to be the same in all samples. Filtering was done to keep genes that reached at least one count per million in at least half of the samples. A heatmap was made for the differentially expressed genes between the high (bad) and low (good) DFI groups using the heatmap function in R (default parameters).

#### Statistical analysis

Normalization was done using the trimmed mean of the *M* values method [33] as implemented in “edgeR”. Moreover, tagwise dispersion was applied to estimate separate mean-variance relationships for the individual genes, and the generalized linear model likelihood test ratio method was employed to test for differential expression. The resulting *p*-values were adjusted for multiple testing by the Benjamini and Hochberg algorithm [34] and the level of significance for differentially expressed genes was set to an false discovery rate (FDR) of 0.05.

### Gene ontology

Gene enrichment analyses make it easier to get an overview of functions that are overrepresented in gene expression datasets. Gene ontology (GO) tools can conveniently assign genes to different terms in the three categories “Molecular Function”, “Cellular Component” and “Biological Process”. In order to map all differentially expressed genes to corresponding GO terms, the R package “goseq” was applied [35]. The Wallenius approximation method was used to account for gene length bias before each GO term was tested for over-representation and under-representation of significant genes. The Benjamini and Hochberg algorithm [34] was used to correct for multiple testing and GO terms were considered significantly enriched at a 0.05 FDR cutoff.

### Variant calling

Variant calling was done within breed using Samtools v.1.1 mpileup and bcftools call [30], and the Integrative Genomics Viewer (IGV) was used to visually inspect putative polymorphisms [36]. Using Samtools v.1.1 bcftools filter, variants (single nucleotide polymorphisms (SNPs)/insertions and deletions (indels)) were filtered to include only those having an alternate allele count of at least two, minor allele frequency above 0.01 and a read depth above 10. Moreover, only polymorphisms in differentially expressed genes were considered. The detected variants were annotated using SnpEff v.4.1 to classify variants (such as missense, nonsense, synonymous, stop gain/loss) and their impact (high, moderate, low, modifier) [37, 38]. Variants causing frameshift mutations or affecting start or stop codons are considered to have high impact, whereas variants e.g. in 3’UTR get the lowest impact (modifier). SnpSift was used to extract relevant information from list of variants files [39]. SNP validation was performed *in-silico* by matching putative polymorphism positions to known pig dbSNP entries [40]. SNPs not present in the database were considered novel. The putative variants identified in differentially expressed genes of this study have been deposited to the European Nucleotide Archive (EVA) under accession number PRJEB22189. For validation purposes, 15 of the high impact variants were genotyped using the KASP SNP genotyping system platform (KBiosciences, Herts, UK) using the 20 animals from the RNA-seq as well as 18 other pigs from Norsvin’s boar testing station (nine from each breed), which are relatives to the RNA-seq boars. SNP validation was also performed in an independent next generation sequencing dataset of related boars [41]. The putative high impact variants were compared by sequence position, reference and alternate alleles to polymorphisms identified in this dataset. Corresponding variants were considered validated.

## Results

### Mapping

Gene expression in testis tissue from Landrace and Duroc boars, with high and low sperm DFI, was analyzed by transcriptome sequencing. The mean (± SD) of the DFI values for the low and high groups were 1.04% (± 0.44%; *n* = 5) and 6.80% (± 1.12%; *n* = 4) in Landrace and 1.09% (± 0.03%; n = 5) and 4.79% (± 0.62%; *n* = 6) in Duroc, respectively (Table 1). The sequence data was maximum 50 basepair reads and the total number of sequenced reads per animal ranged from 59.6 to 95.0 million of which on average 76.7% of the reads were uniquely mapped to the current porcine genome assembly (*Sus scrofa* build 10.2). Altogether, 22,059 genes in Landrace and 21,717 in Duroc had at least one count in at least one sample. After filtering, 14,609 (66.2%) and 14,713 (67.7%) genes were used for differential expression analysis in Landrace and Duroc, respectively.

### Differential expression

A total of 308 genes in Landrace and 374 genes in Duroc were significantly differentially expressed in testis tissue from boars with high and low sperm DFI [see Additional file 1 and Additional file 2 for Landrace and Duroc, respectively]. Of these genes, 71 were common for the two breeds (Table 2). The most significant differentially expressed gene in Landrace and Duroc was actin *ACTA1* (FDR = 2.89e-09 and logarithmic fold change (logFC) = −1.78) and serum amyloid precursor *SAA4* (FDR = 1.90e-06 and logFC = −0.68), respectively. In Landrace, *ACTA1* was also the most down-regulated gene in the high DFI group, whereas neurexophilin *NXPH2* showed the highest up-regulation (FDR = 6.11e-04 and logFC = 3.44). In Duroc, L-dopachrome tautomerase *DCT* showed the most down-regulation (FDR = 2.65e-02 and logFC = −0.94), whereas metallopeptidase *ADAMTS4* was most significantly up-regulated (FDR = 1.88e-02 and logFC = 2.60). The majority of differentially expressed genes (94% and 78% in Landrace and Duroc, respectively) showed increased expression in the high DFI group compared to the low DFI group [see Additional file 5]. In addition to the annotated genes described below, genes encoding functionally uncharacterized proteins were differentially expressed in both breeds and they are included in the results tables with their corresponding Ensembl ID.

**Table 2:** Differentially expressed genes common for the two breeds Landrace and Duroc

Gene symbol	Gene name	FDR L	FDR D
ACER2	alkaline ceramidase 2	1.61E-02	3.74E-02
ACTN4	alpha-actinin	1.52E-02	1.34E-02
APP	amyloid beta A4 protein	2.73E-02	1.34E-02
ATG4A	autophagy related 4A, cysteine peptidase	4.02E-03	2.13E-02
BGN	Biglycan	2.64E-02	1.88E-02
BMP1	bone morphogenetic protein 1	4.60E-02	1.09E-02
SERPING1	Serpin family G member 1	2.68E-02	1.89E-02
GLMP	Glycosylated lysosomal membrane protein	3.66E-03	3.59E-02
C1R	complement component 1, r subcomponent	4.29E-02	2.13E-02
C4A	*Sus scrofa* complement C4 (C4), mRNA	2.86E-02	1.84E-03
CA4	carbonic anhydrase IV	4.06E-02	1.44E-02
CAT	Catalase	1.01E-02	1.24E-02
CDC42EP1	CDC42 effector protein (Rho GTPase binding) 1	4.93E-02	2.42E-02
CITED1	Sus scrofa Cbp/p300-interacting transactivator	2.31E-02	1.11E-02
ENSSSCG00000001711	Uncharacterized protein	3.89E-02	2.72E-02
COL3A1	collagen, type III, alpha 1	4.40E-02	3.59E-02
COPZ2	coatomer protein complex, subunit zeta 2	1.04E-02	4.75E-02
CPED1	cadherin-like and PC-esterase domain containing 1	4.51E-03	2.72E-02
CSF1R	colony stimulating factor 1 receptor	3.86E-02	2.92E-02
CTDSPL	CTD small phosphatase-like	1.52E-02	1.66E-02
CTSA	cathepsin A	2.43E-02	2.71E-02
CTSB	Sus scrofa cathepsin B (CTSB)	1.26E-02	9.65E-03
CTSH	Sus scrofa cathepsin H (CTSH), mRNA	2.07E-02	3.84E-02
CYP11A1	Sus scrofa cytochrome P450, family 11, subfamily A, polypeptide 1	1.58E-02	4.56E-02
ENSSSCG00000028912	Uncharacterized protein	3.02E-02	2.72E-02
CFD	Complement factor D	4.02E-02	1.98E-02
DNASE1L1	deoxyribonuclease I-like 1	4.51E-03	3.59E-02
ECHDC3	enoyl CoA hydratase domain containing 3	1.21E-03	2.04E-02
ENSSSCG00000024587	Uncharacterized protein	2.49E-02	2.64E-02
ENSSSCG00000028244	Uncharacterized protein	2.41E-02	4.91E-02
EDNRA	endothelin receptor type A	4.56E-02	2.42E-02
EFEMP2	EGF containing fibulin-like extracellular matrix protein 2	4.86E-02	1.66E-02
EHD2	EH-domain containing 2	9.26E-03	1.20E-02
ENSSSCG00000011239	Uncharacterized protein	3.85E-02	9.09E-03
ENSSSCG00000021406	Uncharacterized protein	1.35E-02	4.85E-02
ENSSSCG00000025934	Uncharacterized protein	2.75E-02	7.72E-03
ENSSSCG00000029074	Uncharacterized protein	1.61E-02	3.58E-02
EPHX1	Sus scrofa epoxide hydrolase 1, microsomal (xenobiotic) (EPHX1)	1.61E-02	1.11E-02
FAH	fumarylacetoacetate hydrolase (fumarylacetoacetase)	1.66E-02	8.16E-03
FAM213A	family with sequence similarity 213, member A	4.93E-02	1.01E-02
FCGRT	Sus scrofa Fc fragment of IgG, receptor, transporter, alpha	3.42E-02	4.56E-02
FDXR	NADPH:adrenodoxin oxidoreductase, mitochondrial	1.61E-02	1.29E-02
FGFR1	Fibroblast growth factor receptor 1	4.69E-02	1.21E-02
ENSSSCG00000022236	Uncharacterized protein	4.02E-02	1.52E-02
ENSSSCG00000002797	Uncharacterized protein	4.29E-02	2.22E-02
GRK5	G protein-coupled receptor kinase 5	4.60E-02	2.87E-03
GSDMD	gasdermin D	4.11E-02	1.36E-02
ENSSSCG00000000620	Uncharacterized protein	2.01E-02	3.84E-03
ITM2C	Sus scrofa integral membrane protein 2C	4.71E-02	4.75E-02
ENSSSCG00000004207	Uncharacterized protein	3.99E-02	2.36E-02
LAMB2	laminin, beta 2 (laminin S)	1.23E-02	2.81E-02
LAMC3	laminin, gamma 3	1.30E-02	2.15E-02
LIPA	lipase A, lysosomal acid, cholesterol esterase	4.50E-02	3.84E-02
ENSSSCG00000023235	Uncharacterized protein	2.63E-02	1.23E-02
MAOB	monoamine oxidase B	3.62E-02	2.50E-02
NEU1	sialidase 1 (lysosomal sialidase)	4.11E-02	4.56E-02
ENSSSCG00000022516	Uncharacterized protein	2.23E-02	4.97E-02
PGAM1	phosphoglycerate mutase 1 (brain)	1.50E-02	1.29E-02
PLCB1	phospholipase C, beta 1 (phosphoinositide-specific)	4.06E-02	4.75E-02
PRDX2	peroxiredoxin 2	4.02E-02	4.69E-02
ENSSSCG00000016522	Uncharacterized protein	5.88E-03	1.20E-02
SERTAD1	SERTA domain containing 1	3.22E-02	4.35E-02
ENSSSCG00000011357	Uncharacterized protein	3.87E-02	4.11E-02
SLC1A5	solute carrier family 1 (neutral amino acid transporter), member 5	4.99E-02	3.91E-02
SLC41A1	solute carrier family 41 (magnesium transporter), member 1	4.29E-02	7.39E-03
SLC44A2	Choline transporter-like protein 2	4.02E-02	1.12E-02
SLC44A4	Sus scrofa solute carrier family 44, member 4 (SLC44A4), mRNA	4.98E-02	3.50E-02
TMEM176B	transmembrane protein 176B	4.40E-02	7.46E-03
TNFAIP3	tumor necrosis factor, alpha-induced protein 3	2.64E-02	5.56E-03
TPM4	tropomysin alpha-4 chain	5.77E-03	3.10E-02
VIM	Vimentin	4.02E-02	1.12E-02

Genes differentially expressed in both breeds are presented with gene symbol, gene name and significance level (FDR) for Landrace (L) and Duroc (D)

### Gene ontology

**Fig. 1:**
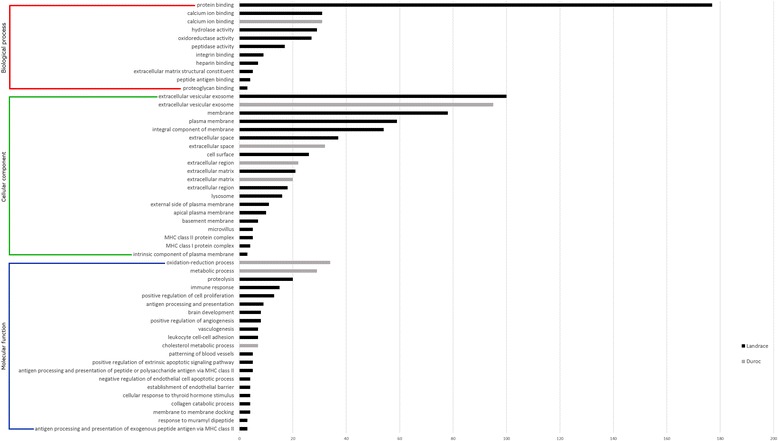
Gene ontology classification of the differentially expressed genes. The figure shows the GO enrichments of the differentially expressed genes in terms of the biological process, cellular component and molecular function classes

Functional characterization of differentially expressed genes revealed an overrepresentation of genes with roles in the cellular components “extracellular matrix” and “extracellular region” for both Landrace and Duroc. Results of the GO classification of the differentially expressed genes are shown in Fig. 1. The molecular function “calcium ion binding” was also enriched in both breeds. In addition, “cholesterol metabolic process” and “oxidation-reduction process” were Duroc specific whereas “collagen catabolic process”, “hydrolase activity” and “proteolysis” were Landrace specific. Moreover, immune system ontologies were Landrace specific.

### Variant calling

Variant detection identified 1501 and 1751 putative polymorphisms in differentially expressed genes in Landrace and Duroc, respectively, out of which 91 and 88% had an existing dbSNP entry [see Additional files 3 and 4]. In Landrace/Duroc, most of the polymorphisms (610/731) in differentially expressed genes were synonymous SNPs (Table 3). Of the polymorphisms in differentially expressed genes, 4/17 in Landrace/Duroc were high impact variants, predicted to cause frameshifts or a change in start or stop codon. 15 of the high impact variants were chosen for validation using the KASP SNP Genotyping system. Five of the SNPs were successfully validated, including four of the ones with previous dbSNP entries (see Additional file 7]. Ten of the detected high impact variants, including one with an existing dbSNP entry, failed validation. When comparing the variants to an independent next generation sequencing dataset, the same result was found. The differentially expressed genes with validated high impact variants were *RAMP2*, *GIMAP6, ENSSSCG00000000712*, *ENSSSCG00000009348* and *ENSSSCG00000028326.*

**Table 3:** Effects of putative SNPs

SNP effect	After filtration		Joined with gene expression results	
	Landrace	Duroc	Landrace	Duroc
3’UTR	17,741	17,715	573	627
5’UTR	3622	3691	62	102
Frameshift	453	516	3	12
Missense	9946	10,424	267	246
Splice acceptor	16	17	0	0
Splice donor	12	12	0	0
Splice site region	338	362	3	7
Start lost	13	8	1	0
Stop gained	68	63	0	3
Stop lost	27	34	0	2
Synonymous	21,060	21,273	610	731
SNP impact				
High	600	663	4	17
Moderate	10,006	10,486	269	250
Low	21,974	22,200	623	752
Modifier	23,251	23,293	676	807

SNP effect according to SnpEff for putative polymorphisms detected in Landrace and Duroc. The results presented are after filtration and joined with differentially expressed genes. Some SNPs have more than one predicted effect

## Discussion

Chromatin condensation and DNA packaging in sperm cells occur during testicular spermatogenesis, and altered chromatin structure is associated with sperm DFI. High levels of sperm DFI has been associated with decreased fertility, however, the molecular mechanisms contributing to alterations in sperm DFI is not clear. In the present study, we explored gene expression differences in testis between groups of boars with high and low sperm DFI and investigated the gene enrichments associated with the results. The experiment was performed in two different breeds, Landrace and Duroc, and 308 and 374 genes were found differentially expressed in Landrace and Duroc, respectively. Of these genes, 71 were found to be common for the two breeds, which means they are likely to be essential for alterations in sperm DFI. The Landrace specific and Duroc specific differentially expressed genes might reflect breed specific mechanisms in chromatin condensation and DFI level with regards to these two breeds. Breed differences in DFI have also previously been found in boars as well as bulls [42, 43]. The GO terms “extracellular matrix”, “extracellular region” and “calcium ion binding” were significant for both breeds and differentially expressed genes belonging to these pathways are discussed in more detail below. None of the differentially expressed pathways were found to overlap with pathways previously identified for spermatogenesis in Large White, Duroc and Meishan pigs [44, 45], indicating that we have identified pathways related to DFI and not general spermatogenesis.

### Genes enriched in “extracellular matrix” and “extracellular region”

The seminiferous tubules in testis contain Sertoli and germ cells and direct progression of spermatogenesis. The “extracellular matrix”, an enriched GO term in both Landrace and Duroc, plays a significant role in regulating spermatogenesis because Sertoli and germ cells are structurally and hormonally supported by extracellular matrix during their development in the seminiferous tubules [46]. To complete spermatogenesis, germ cells must migrate across the seminiferous epithelium while still attached to the nourishing Sertoli cells, a process controlled by restructuring events at cell junctions known as ectoplasmic specialization [46, 47]. This is the stage where DNA compaction and chromatin condensation occur [48]. These junctions are located in the “extracellular region” [47], another enriched GO term in both breeds. The results suggest that genes involved in different stages of spermatogenesis affect DNA fragmentation in sperm cells.

Laminins and collagens are important building blocks of the extracellular matrix in testis and they act together with proteases, protease inhibitors, cytokines and focal adhesion components to regulate membrane proteins [46]. Two genes of the laminin family (*LAMB2* and *LAMC3*) and one of the collagen family (*COL3A1*) were found up-regulated in the high DFI group in both breeds in this study. Both pre-collagens and laminins are processed by bone morphogenetic protein 1 (*BMP1*) [49], which was also up-regulated in the high DFI condition in both breeds. Furthermore, genes of the collagen family were exclusively up-regulated in the high DFI group in one of the breeds (*COL1A1* in Duroc and *COL1A2, COL4A1, COL4A2* and *COL14A1* in Landrace). The differential expression of the laminin and collagen genes might suggest that the structure of the extracellular matrix, where the sperm cells are attached during development, could influence chromatin condensation and hence DFI level. This is also supported by the differential expression of genes encoding other components of the extracellular matrix such as the cytokines tumor necrosis factor (TNF) alpha and interleukins. TNFα regulates germ cell apoptosis, Leydig cell steroidogenesis and junction dynamics in the testes [46] and it has also been shown to induce sperm damage such as DNA fragmentation [50]. TNF member *TNFAIP3* was up-regulated in the high DFI group in both breeds in this study. Additionally, breed specific up-regulation in the high DFI group was found for genes of this family (*TNFSF10* and *TNFRSF12A* in Landrace and *LITAF* in Duroc). Interleukin *IL1R1* was up-regulated in the high DFI group in Landrace. This is in agreement with previous findings, where IL1R1 protein was associated with DFI in human sperm and seminal plasma [18, 19].

Genes encoding fibulins, proteases, protease inhibitors and cathepsins, all interacting with components of the extracellular matrix, were also differentially expressed in this study. Fibulins are extracellular matrix glycoproteins that modulate cellular behavior and function and are involved in binding of laminin and calcium [51, 52]. In this study EGF containing fibulin-like EM protein 2 (*EFEMP2*, also known as *FBLN4*) was up-regulated in the high DFI group in both breeds whereas fibulins *FBLN5* and *EFEMP1* (also known as *FBLN3*) were up-regulated in Duroc. Furthermore, extracellular matrix protein 1 (*ECM1*), known to interact with fibulins and laminins [53], was up-regulated in Duroc. The ECM1 protein has previously been found associated with sperm DNA fragmentation in human seminal plasma [19], supporting the findings of this study. Matrix metallopeptidases (MMPs) and MMP inhibitors (TIMPs) are proteases and protease inhibitors, respectively. They are capable of degrading different components of the extracellular matrix, like laminins and collagen, and thereby regulate spermatogenesis [46, 54]. A disintegrin and metalloproteases (ADAMs) regulate spermatogenesis by cleaving growth factors and cytokines from the extracellular matrix [54]. In this study, *MMP2*, *MMP19, TIMP1* and *ADAMTS9* were up-regulated in the high DFI group in Landrace whereas *TIMP3, ADAM33* and *ADAMTS4* were up-regulated in Duroc. *ADAMTS4* was the most up-regulated gene in Duroc in this study indicating an important role for proteases in DNA fragmentation of sperm cells, possibly by interrupting with the testicular extracellular matrix stability. Cathepsins contribute in protein degradation in the extracellular matrix by cleaving collagens and laminins [55]. The cathepsin members *CTSA, CTSB* and *CTSH* were found up-regulated in the high DFI group of both breeds. Additionally, *CTSC, CTSL* and *CTSS* were up-regulated in Landrace. Interestingly, *CTSL* has been linked to chromatin decondensation in sea urchin embryos [56] and CTSA has been shown to affect sperm motility in rats [57]. Moreover, CTSB, CTSC, CTSD, CTSL and CTSS are all involved in testis tissue restructuring during spermatogenesis in rats [58].

Peroxiredoxins are located in the ectoplasmic specialization and encode redox proteins, which protect sperm cells from oxidative stress that cause DNA damage such as DNA fragmentation [59]. In this study, peroxiredoxin *PRDX2* was up-regulated in the high DFI group in both breeds whereas *PRDX3* was up-regulated in the high DFI group in Duroc. Furthermore, glutathione peroxidase *GPX3* was up-regulated in the high DFI group in Landrace. These results are supported by previous findings in human, where levels of peroxiredoxin members PRDX1 and PRDX6 have been associated with sperm DNA integrity [60]. The differentially expressed gene *GPX3* is interesting since glutathione peroxidases can work both as redox proteins and to mediate disulfide bridging, which stabilizes sperm chromatin [61].

Actins are important components of the ectoplasmic specialization of the seminiferous tubules [46, 47] and are involved in the development of mature sperm through several processes, including chromatin remodeling [62, 63]. The *ACTN4* was up-regulated in high DFI boars of both breeds. In Landrace, three additional actin and actin-binding proteins were found to be differentially expressed (*ABLIM1, ACTA1* and *ACTA2*). *ACTA1* was down-regulated in the high DFI group, whereas the other actin members were up-regulated, indicating different functions of these actin members when developing DFI in the testis. It was the most down-regulated of the differentially expressed genes in Landrace, suggesting an important role for this gene in DFI levels of this breed. In Duroc, coronin acting binding protein 1B (*CORO1B*) and demantin actin binding protein (*DMTN*) were up-regulated whereas capping protein (actin filament) muscle Z-line, alpha 3 (*CAPZA3*) was down-regulated. The significance of different actin genes between the two breeds could imply breed specialized mechanisms, however, this needs to be further investigated.

In this study, genes encoding extracellular matrix compounds such as collagens, laminins, fibulins and cytokines were differentially expressed. Moreover, peroxiredoxins and actins of the ectoplasmic specialization were up- and down-regulated. Genes involved in regulation of these compounds, like proteases, protease inhibitors and cathepsins, were also differentially expressed. The results confirm previous findings, as well as reporting a number of new genes, highlighting the importance of testicular steroidogenesis in the outcome of sperm DFI. In this study, a major part of the differentially expressed genes were up-regulated. A hypothesis explaining this could be that deficiencies of the extracellular matrix makes the cell compensate by up-regulating gene expression.

### Genes enriched in “calcium ion binding”

The GO term calcium ion binding was significantly enriched in both breeds and calcium uptake in sperm is known to be important for the regulation of fertility by affecting sperm maturation, motility, capacitation and the acrosome reaction [64, 65]. A role for calcium in chromatin condensation and DFI is less described, however, the calcium permeable ion channels proteins VDAC2 and VDAC3 have previously shown significant association with DFI in human sperm [18] and fertility in boars [24]. Moreover, along with chromatin condensation in spermatogenesis, the sperm cell redundant nuclear envelope evolves, which has been proposed a role in calcium ion storage [64]. This could explain the significance of the “calcium ion binding” enrichment in both breeds of this study.

The up-regulation of voltage-dependent anion channel gene *VDAC1* in the high DFI group in Duroc is interesting as the proteins VDAC2 and VDAC3 has been associated with DNA fragmentation in human sperm [18]. Moreover, abnormal regulation of different calcium channels has previously been shown to negatively affect sperm function [66]. A number of other genes involved in reproduction related processes where calcium influx plays a role, like hyperactivation, capacitation, the acrosome reaction and fertilization [63], were differentially expressed in this study (*PLCB1* in both breeds*, PLCZ1, DLD* and *PLD1* in Duroc, and *PDGFRB*, *CAPN1*, *PLA2G4A*, *NPR1* and *RAPGEF3* in Landrace). The up-regulation of all these genes in boars with high sperm DFI could imply an interrupted function of calcium mediated regulation, which would affect the fertilizing capability of these sperm cells after being ejaculated. Further studies are needed, however, to clarify the role of testicular calcium signaling in sperm DFI levels.

### Variant detection

An advantage of calling genomic variants from transcriptome sequencing data is that it directly allows for detection of polymorphisms in transcribed regions and is an efficient way to discover putative causative SNPs. Variant detection requires sufficient coverage with high quality sequence reads in order to distinguish true polymorphisms from sequencing errors. Filtering on sequencing depth might have removed polymorphisms in low expression genes, however, visualization by IGV showed likely false positive variants if this filtering was not done. This is in agreement with another study showing that the majority of false positive SNPs occur at sites with less than 10X coverage [67]. Comparing our detected polymorphisms with variants in dbSNP showed that 91 and 88% of our putative polymorphisms in Landrace and Duroc had a corresponding dbSNP entry, respectively. However, only five of the predicted high impact variants had an existing dbSNP entry and a validation study was therefore conducted to test 15 of the putative high impact SNPs. The results showed that high impact variants in the differentially expressed genes *RAMP2*, *GIMAP6, ENSSSCG00000000712*, *ENSSSCG00000009348* and *ENSSSCG00000028326* are particularly interesting for sperm DNA fragmentation in boars. Failure to validate ten of the variants shows that SNP detection in short read sequencing data can produce false positives. It has been shown that a number of factors can contribute to false positive SNPs in sequence data, including quality of the reference sequence, read length, choice of mapper and variant caller, mapping stringency and filtering of SNPs [68]. The importance of a high quality reference genome was highlighted in Ribeiro et al. (2015) [68] and we know that the reference genome used in this study has its limitations [69]. Approximately 90% overlap of our identified SNPs and previously identified SNPs does however indicate that our pipeline works, but that caution should be taken especially for variants with no supporting evidence. The identical results of validation using a PCR-based method (KASP) and in silico in an independent dataset could suggest that the latter is equally good in those cases where datasets are available.

Although many of the putative polymorphisms identified are located outside open reading frames or cause synonymous changes, they may be in linkage disequilibrium to other causative mutations. Moreover, studies have also shown that synonymous SNPs may have functional effects by affecting mRNA stability or by translation suppression [70, 71].

## Conclusions

The present study identified whole genome expression differences in testis tissue between boars with high and low levels of sperm DFI. Moreover, putative polymorphisms were detected in the differentially expressed genes. The results of this study show that differentially expressed genes of steroidogenic pathways, where the chromatin condensation and DNA packaging occurs, are important for the outcome of DFI levels in ejaculated spermatozoa. Transcriptome sequencing analysis showed that the major changes at transcription level in the testicle of pig concerning sperm DFI were related to the functional categories “extracellular matrix”, “extracellular region” and “calcium ion binding”. Variant detection showed that predicted high impact SNPs in *RAMP2*, *GIMAP6* and three uncharacterized genes are particularly interesting for the trait. The candidate genes identified in this study provide a valuable resource to identify molecular markers for sperm DFI, for use in selection towards improved sperm quality.

## Additional files

Additional file 1: Differentially expressed genes for DFI in Landrace. The results are presented with Ensembl gene id, gene symbol, gene name, fold change and significance level (FDR). (XLSX 31 kb)

Additional file 2: Differentially expressed genes for DFI in Duroc. The results are presented with Ensembl gene id, gene symbol, gene name, fold change and significance level (FDR). (XLSX 34 kb)

Additional file 3: High quality SNPs occurring in differentially expressed genes in Landrace. The SNPs are presented with Ensembl gene id, gene name, FDR value of differentially expressed gene, chromosome (SSC), position, reference allele and alternate allele, as well as effect, impact according to SnpEff and dbSNP ID. (XLSX 100 kb)

Additional file 4: High quality SNPs occurring in differentially expressed genes in Duroc. The SNPs are presented with Ensembl gene id, gene name, significance level (FDR) of differentially expressed gene, chromosome (SSC), position, reference allele and alternate allele, as well as effect, impact according to SnpEff and dbSNP ID. (XLSX 117 kb)

Additional file 5: Heatmap of the differentially expressed genes for DFI. The differentially expressed genes in testis of A) Duroc and B) Landrace boars with high (bad) and low (good) sperm DFI ordered by hierarchical clustering show higher (red) and lower (yellow) expression of genes in the two DFI groups. (TIFF 59 kb)

Additional file 6: The edgeR source code used for testing for differential expression. (TXT 2 kb)

Additional file 7: Putative high impact SNPs in differentially expressed genes. Putative high impact SNPs in differentially expressed genes presented with breed, gene name, position, FDR and log fold change. Validation by KASP SNP Genotyping System (N.A. is for SNPs not tested). (DOCX 12 kb)

## Abbreviations

ABLIM1: Actin binding LIM protein 1
ACTA1: Actin, alpha 1, skeletal muscle
ACTA2: Actin, alpha 2, smooth muscle, aorta
ACTN4: Actinin alpha 4
ADAM33: ADAM metallopeptidase domain 33
ADAMs: A disintegrin and metalloproteases
ADAMTS4: ADAM metallopeptidase with thrombospondin type 1 motif, 4
ADAMTS9: ADAM metallopeptidase with thrombospondin type 1 motif, 9
AI: Artificial insemination
AO: Acridine orange
BMP1: Bone morphogenetic protein 1
CAPN1: Calpain 1
CAPZA3: Capping actin protein of muscle Z-line alpha subunit 3
COL14A1: Collagen type XIV alpha 1 chain
COL1A1: Collagen type I alpha 1 chain
COL1A2: Collagen type I alpha 2 chain
COL3A1: Collagen type III alpha 1 chain
COL4A1: Collagen type IV alpha 1 chain
COL4A2: Collagen type IV alpha 2 chain
CORO1B: Coronin 1B
CTSA: Cathepsin A
CTSB: Cathepsin B
CTSC: Cathepsin C
CTSH: Cathepsin H
CTSL: Cathepsin L
CTSS: Cathepsin S
DCT: Dopachrome tautomerase
DFI: DNA fragmentation index
DLD: Dihydrolipoamide dehydrogenase
DMTN: Dematin actin binding protein
ECM1: Extracellular matrix protein 1
EFEMP1: EGF containing fibulin-like extracellular matrix protein 1
EFEMP2: EGF containing fibulin-like extracellular matrix protein 2
ERCC1: Excision repair cross-complementing gene 1
EV: Electronic volume
FBLN4: Fibulin 4
FBLN5: Fibulin 5
FDR: False discovery rate
GEO: Gene expression omnibus
GIMAP6: GTPase, IMAP family member 6
GO: Gene ontology
GPX3: Glutathione peroxidase 3
IGV: Integrative genomics viewer
IL1R1: Interleukin 1 receptor type 1
Indel: Insertion/deletion
LAMB2: Laminin, beta 2
LAMC3: Laminin, gamma 3
LITAF: Lipopolysaccharide-induced TNF
logFC: Logarithmic fold change
MMP19: Matrix metallopeptidase 19
MMP2: Matrix metallopeptidase 2
MMPs: Matrix metallopeptidases
NPR1: Natriuretic peptide receptor 1
NXPH2: Neurexophilin 2
PDGFRB: Platelet-derived growth factor receptor beta
PLA2G4A: Phospholipase A2 group IVA
PLCB1: Phospholipase C beta 1
PLCZ1: Phospholipase C zeta 1
PLD1: Phospholipase D1
PRDX2: Peroxiredoxin 2
PRDX6: Peroxiredoxin 6
RAMP2: Receptor activity modifying protein 2
RAPGEF3: Rap guanine nucleotide exchange factor 3
RIN: RNA integrity number
SAA4: Serum amyloid A4, constitutive
SCSA: Sperm chromatin structure assay
SD: Standard deviation
SNP: Single nucleotide polymorphism
SS: Side scatter
TIMP1: TIMP metallopeptidase inhibitor 1
TIMP3: TIMP metallopeptidase inhibitor 3
TIMPs: MMP inhibitors
TNF: Tumor necrosis factor
TNFAIP3: Tumor necrosis factor, alpha-induced protein 3
TNFRSF12A: TNF receptor superfamily member 12A
UTR: Untranscribed region
VDAC1: Voltage dependent anion channel 1
VDAC2: Voltage dependent anion channel 2
VDAC3: Voltage dependent anion channel 3
