# Supplementary figures and images for: RNA sequencing reveals candidate genes and polymorphisms related to sperm DNA integrity in testis tissue from boars
Source: BMC Vet Res. 2017 Nov 28;13:362. doi: 10.1186/s12917-017-1279-x (PMC5706377; doi:10.1186/s12917-017-1279-x)

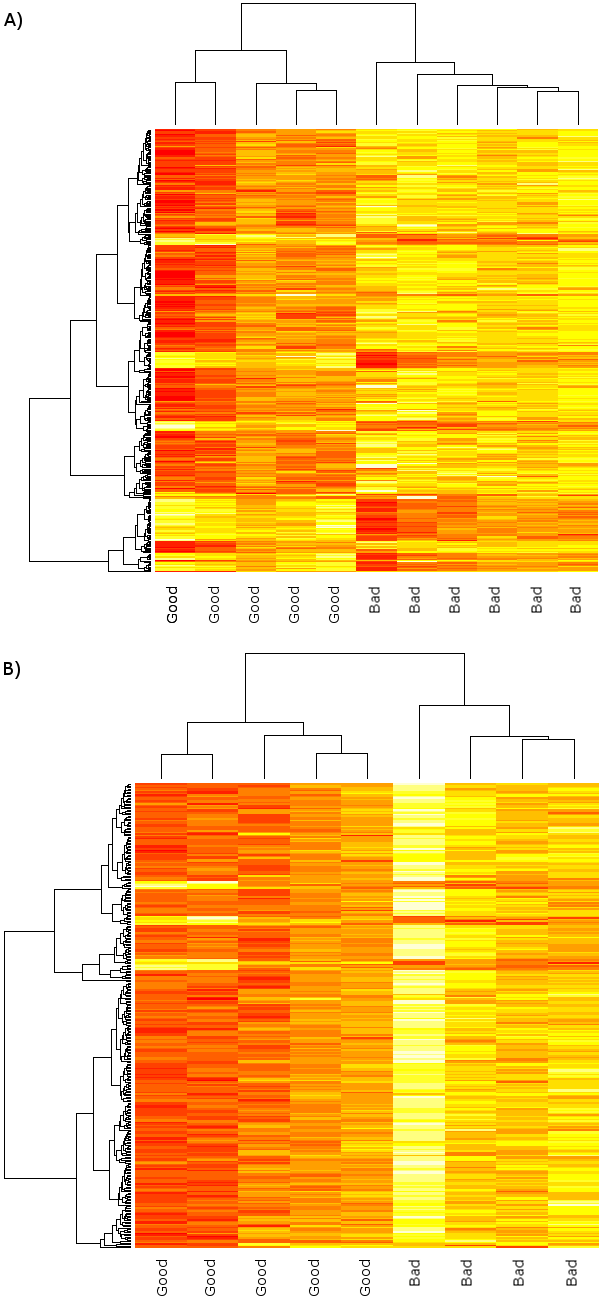

Supplement: Supplementary file 5 — Heatmap of the differentially expressed genes for DFI. The differentially expressed genes in testis of A) Duroc and B) Landrace boars with high (bad) and low (good) sperm DFI ordered by hierarchical clustering show higher (red) and lower (yellow) expression of genes in the two DFI groups. (TIFF 59 kb) [file 12917_2017_1279_MOESM5_ESM.tiff]
