# Supplementary material for: RNA sequencing reveals candidate genes and polymorphisms related to sperm DNA integrity in testis tissue from boars
Source: BMC Vet Res. 2017 Nov 28;13:362. doi: 10.1186/s12917-017-1279-x (PMC5706377; doi:10.1186/s12917-017-1279-x)
Supplement: Supplementary file 7 — Putative high impact SNPs in differentially expressed genes. Putative high impact SNPs in differentially expressed genes presented with breed, gene name, position, FDR and log fold change. Validation by KASP SNP Genotyping System (N.A. is for SNPs not tested). (DOCX 12 kb) [file 12917_2017_1279_MOESM7_ESM.docx]

| Breed | Gene | Chromosome | SNP position | FDR | LogFC | KASP |
| --- | --- | --- | --- | --- | --- | --- |
| Landrace | ENSSSCG00000000712 | 5 | 67005497 | 6.77E-03 | 1.20 | Ok |
| Landrace | RAMP2 | 12 | 20347139 | 1.19E-02 | 1.19 | Ok |
| Landrace | BMP1 | 14 | 6887020 | 4.56E-02 | 0.77 | Fail |
| Landrace | ENSSSCG00000012925 | 2 | 4576337 | 4.71E-02 | 1.72 | Fail |
| Duroc | ALAS1 | 13 | 37666903 | 1.70E-05 | 1.35 | Fail |
| Duroc | DGKH | 11 | 25503370 | 2.76E-04 | -0.59 | Fail |
| Duroc | GIMAP6 | 18 | 6630873 | 5.96E-04 | 0.87 | Ok |
| Duroc | ENSSSCG00000028326 | GL892233.1 | 1316 | 1.83E-03 | 1.62 | Ok |
| Duroc | USP25 | 13 | 190976277 | 2.69E-03 | -0.46 | Fail |
| Duroc | BMP1 | 14 | 6887020 | 1.09E-02 | 0.48 | Fail |
| Duroc | KIF21A | 5 | 73414065 | 1.36E-02 | 0.42 | N.A. |
| Duroc | ENSSSCG00000017421 | 12 | 21106727 | 1.58E-02 | 0.47 | N.A. |
| Duroc | ENSSSCG00000017421 | 12 | 21106729 | 1.58E-02 | 0.47 | Fail |
| Duroc | LRP10 | GL896267.1 | 50406 | 1.98E-02 | 0.61 | N.A. |
| Duroc | CBLC | 6 | 47186767 | 2.86E-02 | -0.53 | Fail |
| Duroc | TTC25 | 12 | 21078400 | 3.17E-02 | -0.30 | Fail |
| Duroc | PANK1 | 14 | 110459890 | 3.59E-02 | 1.18 | Fail |
| Duroc | AVEN | GL895866.1 | 14095 | 4.39E-02 | -0.32 | N.A. |
| Duroc | ENSSSCG00000021848 | GL896314.1 | 8938 | 4.87E-02 | -0.28 | Fail |
| Duroc | ENSSSCG00000009348 | 11 | 9432848 | 4.91E-02 | 0.57 | Ok |
| Duroc | ZBBX | 13 | 114805494 | 4.97E-02 | -0.30 | N.A. |
